# Effect of Ethanol Extracts of Propolis (EEPs) against Staphylococcal Biofilm—Microscopic Studies

**DOI:** 10.3390/pathogens9080646

**Published:** 2020-08-11

**Authors:** Katarzyna Grecka, Zirui Ray Xiong, Hanyu Chen, Karolina Pełka, Randy W. Worobo, Piotr Szweda

**Affiliations:** 1 Department of Pharmaceutical Technology and Biochemistry, Faculty of Chemistry, Gdańsk University of Technology, Str. G. Narutowicza 11/12, 80-233 Gdańsk, Poland; karolina.pelka@pg.edu.pl; 2 Department of Food Science, Cornell University, Ithaca, New York, NY 14853, USA; zx97@cornell.edu (Z.R.X.); hc967@cornell.edu (H.C.); rww8@cornell.edu (R.W.W.)

**Keywords:** propolis, *Staphylococcus aureus*, *Staphylococcus epdermidis*, biofilm, fluorescence microscopy, extracellular polymeric substances (EPS)

## Abstract

Staphylococci growing in the form of biofilm exhibit high resistance to a plethora of antibiotics. The aim of the study was to assess the influence of ethanolic extract of propolis (EEPs) on *S. epidermidis* ATCC 35984 biofilm using fluorescent microscopy. Propidium iodide (PI) and SYTO 9 were used for differentiation of live and dead cells, and calcofluor white was used to stain the extracellular matrix, the self-produced extracellular polymeric substances (EPS). The outcomes of the research confirm the promising potential of EEPs for eradication of staphylococcal biofilm. However, its activity cannot be classified as fully satisfactory, either in terms of the effectiveness of elimination of bacterial cells or disturbing the EPS structure. A two or even four times higher concentration of EEPs compared to MIC (Minimum Inhibitory Concentration) against planktonic cells (128 µg/mL) was necessary for effective (estimated for 90%) elimination of living cells from the biofilm structure. Unfortunately, even at that concentration of EEPs, the extracellular matrix was only partially disturbed and effectively protected the residual population of living cells of *S. epidermidis* ATCC 35984. In our opinion, a combination of EEPs with agents disrupting components of EPS, e.g., proteases, lysines, or enzymes degrading extracellular DNA or PIA (polysaccharide intercellular adhesin).

## 1. Introduction

Bacteria of the genus *Staphylococcus*, particularly *S. aureus* and *S. epidermidis*, belong to the most common but also most dangerous human and animals’ pathogens. They produce a broad range of virulence factors, are common in the environment, and easily evolve and acquire resistance to a plethora of antibiotics [1,2,3,4]. Another important feature of these bacteria, both in terms of the frequency of infection and the effectiveness of their treatment, is their ability to grow in the form of biofilm, a community of cells that are embedded in a matrix of self-produced extracellular polymeric substances (EPS) [4,5,6]. The matrix consists mostly of the biofilm mass and is composed of polysaccharides (the most important of them is poly-N-acetylglucosamine, which is also called PIA (polysaccharide intracellular adhesion), proteins, and extracellular nucleic acids [6]. Due to differences in gene expression and protein production, cells in the biofilm exhibit different phenotypes and metabolism features in comparison to planktonic growth. This has been proven for both *S. aureus* and *S. epidermidis* [7,8]. From a clinical point of view, the most important factor that influences the effectiveness of different approaches for prophylaxis and treatment of infections is that staphylococci growing in the form of biofilm can develop many different mechanisms that protect them against immune evasion—phagocyte attacks, antimicrobial agents (e.g., defensins) produced by the infected host, as well as antibiotics and synthetic antimicrobials. Surrounded by the extracellular polymeric substances, cells are “invisible” and physically unavailable for human or animal immune systems. Components of the EPS, e.g., negatively charged DNA or proteins, significantly block host-produced antimicrobial peptides within the biofilm structure. However, the lower activity of antibiotics and other antimicrobial agents of low molecular weight is probably the consequence of the decreased metabolic activity of the bacterial cells growing in the biofilm compared to planktonic cells [5,9]. Some authors revealed 10 to 1000 times lower susceptibility of staphylococcal biofilm to the most common antibiotics [10,11]. Similarly, the outcomes of our recent investigation revealed a much lower susceptibility of *S. aureus* biofilm towards newly synthesized anthra [1–*d*][1,2,3]triazine-4,7,12(3*H*)-triones compared to planktonic cells [12].

For about 30% of humans, *S. aureus* is a part of their normal flora [13,14], and even more people are asymptotic carriers of *S. epidermidis—*an innocuous commensal microorganism on the human skin [15,16,17]. Consequently, these bacteria are the most frequent etiological factors of biofilm infections of wounds and surgically implanted indwelling medical devices, e.g., joint prostheses or catheters, which are extremely hard to cure [9,17]. Biofilm formation is considered an important virulence factor that increases the risk of infection recurrence and treatment failures [5,9,17]. Due to the high resistance of biofilms to classical antibiotic therapy, there is an urgent need to identify new agents that will be effective against staphylococcal biofilms. Some of the most promising strategies for treatment and prophylaxis of staphylococcal biofilm-associated infections have been recently presented in the review of Otto [5]. These strategies include antimicrobial peptides, bacteriophages and lysines, vaccines, biofilm-degrading enzymes, quorum sensing blockers, and coating the surfaces of the medical devices with antimicrobial agents such as silver or copper. Some natural products of plant origin and substances derived from marine organisms, mainly sponges, have been shown to be effective in the eradication of staphylococcal biofilms [18]. Our preliminary research confirmed the high efficiency of ethanolic extract of propolis (EEPs) against staphylococci growing in the form of biofilms. However, significant differences were observed in the activity of the samples, and there was a strong correlation between concentration of phenolic components and the efficacy of the propolis extracts [19]. This current study is a more detailed analysis of the influence of the ethanolic extract of propolis (with confirmed high polyphenols content) on *S. epidermidis* ATCC 35984 biofilm using confocal laser scanning microscopy (CLSM). Propidium iodide (PI) and SYTO 9 were used for differentiation of live and dead cells, and calcofluor white (CFW) was used to stain beta 1-4 linked polymers in EPS. The effectiveness of propolis extract was tested against biofilm growing on polymeric and metal surfaces. Imaging chambers with a coverslip bottom and nano-smooth aluminum oxide coupons were used as the model surfaces for biofilms growth. Aluminum oxide is a material generally recognized as safe (GRAS) and commonly used in the medical industry. Due to its biocompatibility and chemical properties, it is commonly used as a material for orthopedic and dental implants as well as for general hospital and lab equipment. The outcomes of the research confirmed a promising potential of EEPs for staphylococcal biofilms eradication.

## 2. Results

The selection of the propolis sample for microscopic studies was based on the outcomes of two preliminary assays: determination of total phenolic content and determination of MIC values against planktonic cells of selected strains of staphylococci, which were performed according to procedures presented in our previous report [19]. The investigation of Williams and Bloebaum indicated that, after 48 h of growth, the *S. epidermidis* ATCC 35984 produced a mature biofilm with a significant network of matrix components and found this strain particularly useful for microscopic studies of staphylococcal biofilms [20]. The propolis sample used for the preparation of the most promising ethanolic extract was provided by the owners of apiary Miodolandia, which is located in Stanisławowo, near Gdańsk, and collects bee products in the northern region of Poland. The extract produced from this product was rich in polyphenols (125.86 ± 3.2 mg GAE/1g) and exhibited high anti-staphylococcal activity with MIC value of 128 µg/mL against planktonic cells of reference strains of *S. epidermidis* ATCC 35984, *S. epidermidis* ATCC 12228, *S. aureus* ATCC 29213, and *S. aureus* ATCC 25923. The same MIC value was also found for nine clinical isolates of *S. aureus,* including three MRSA strains (Table 1).

**Figure 1:**
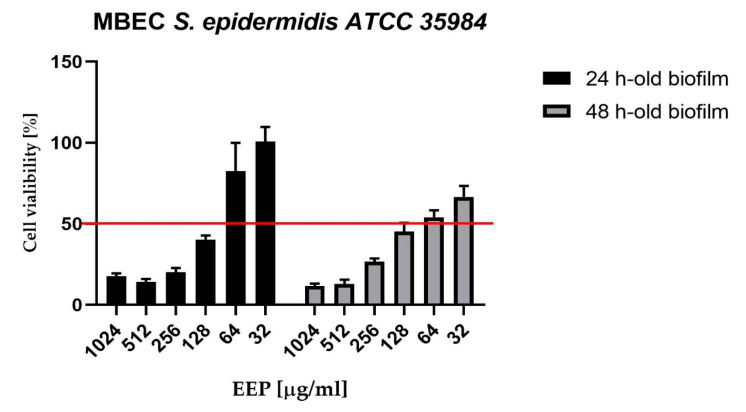
Anti-biofilm activity of EEP against *S. epidermidis* ATCC 35984.

Moreover, we also determined the antibiofilm potential of selected EEP with the “standard” approach—measurement of biochemical activity (MTT reduction) of bacterial cells forming the biofilm structure. The metabolic (MTT) assay was used to assess the viability of 24 and 48 h-old biofilms after treatment with propolis. The MBEC50 (Minimal Biofilm Eradication Concentration that causes a 50% reduction of the metabolic activity of bacterial cells living in the structure of biofilm) value was equal to the MIC (128 µg/mL) in the case of both variants of the experiment (Figure 1). However, increasing the concentration did not reduce the viability by more than 90%. In addition, 48 h-old biofilm was used for microscopic studies as it was found to provide repeatable results and stable formation of rigid biofilm.

**Figure 2:**
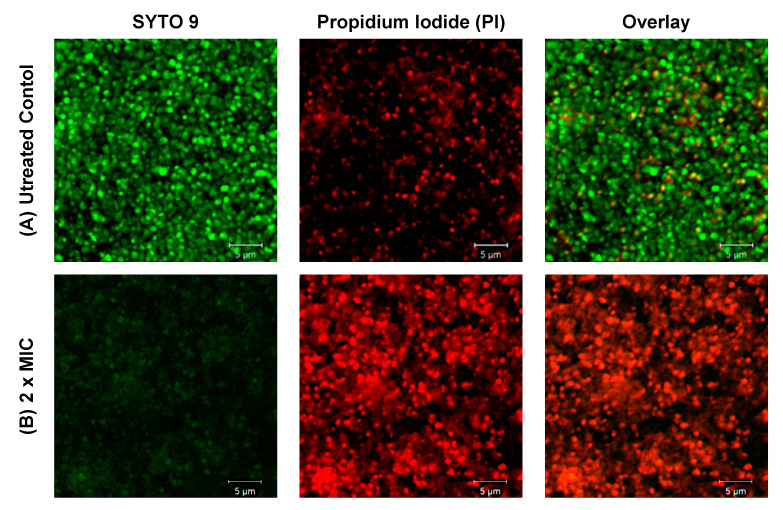
Fluorescence microscopy images of *S. epidermidis* biofilms. (**A**) The untreated control constituted a 48 h-old biofilm further cultivated for 24 h without the presence of EEPs. (**B**) *S. epidermidis* 48 h-old biofilms formed in an 8-well chambered coverslip were treated with an ethanolic extract of propolis (EEPs) at a concentration 2x MIC (256 µg/mL) for 24 h. Bacteria were labeled with LIVE/DEAD BacLight SYTO 9 (green) and PI (red). Scale bar = 5 μm.

**Figure 3:**
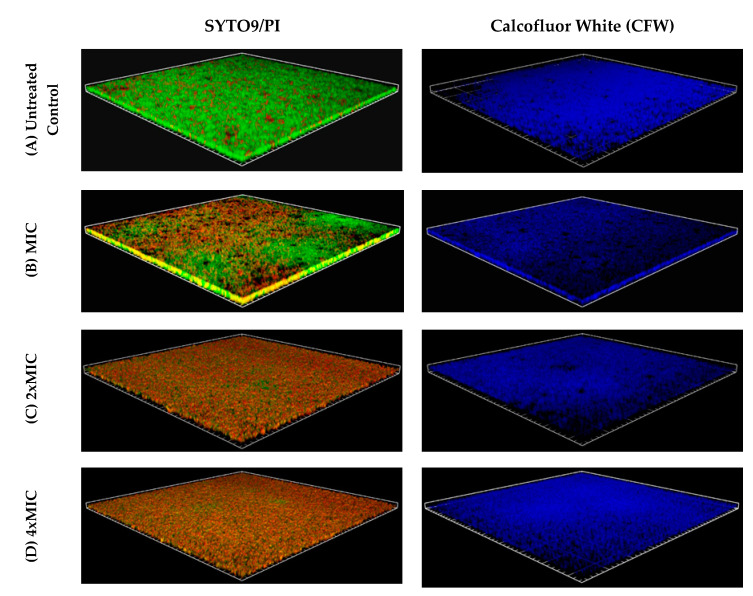
Confocal laser scanning microscopy images of *S. epidermidis* biofilms formed in an 8-well chambered coverslip treated with different concentrations of propolis extract. (**A**) The untreated control constituted a 48 h-old biofilm further cultivated for 24 h without the presence of EEPs. (**B**) *S. epidermidis* 48 h-old biofilms were treated with ethanolic extract of propolis (EEPs) at concentrations (**B**) MIC (128 µg/mL), (**C**) 2x MIC and (**D**) 4x MIC for 24 h. The first column displays bacteria labeled with LIVE/DEAD BacLight SYTO 9 (green) and PI (red) and the second column EPS labeled with CFW (blue). Scale units (grids) are 40 µm in length.

The differences in the result of staining of control (untreated) and affected with EEP (at a concentration of 2 x MIC) biofilm are presented in Figure 2 (2D picture). It is clearly shown that some of the staphylococcal cells within the structure of biofilm are killed by the components of propolis. The propidium iodide is a red-fluorescent DNA-specific stain that penetrates only cells with disrupted membranes, which is why it is used for the identification of dead cells. As it is shown in Figure 3 (3D pictures), at the concentration equal to MIC against planktonic cells (128 µg/mL), only partial elimination of living cells from the biofilm structure was achieved. Stronger antagonistic effects were observed at concentrations of 2 or 4x MIC (256 and 512 µg/mL, respectively). In both cases, the number of green—living—cells was much lower in comparison to the biofilm treated with EEPs at the MIC concentration. This result clearly confirms that antibacterial components of EEPs effectively penetrate the structure of the biofilm and inactivate the bacteria, and only slightly higher resistance was observed compared to planktonic cells. However, careful analysis of the 3D picture of the biofilm treated with EEPs at a concentration of 4xMIC (Figure 3) clearly indicates that some living (green) cells still remain within the structure of biofilm. This might be a very important observation from the clinical point of view. This residual cell population may be the reason for the recurrence of the diseases. Interesting conclusions come from the analysis of Figure 3C,D. In these pictures, the EPS stained with calcofluor white is presented. In contrast to the bactericidal effect, which was quite good, it can be estimated that more than 90% of cells were eliminated. Even the highest applied concentration 4x MIC only partially disturbed the extracellular matrix.

**Figure 4:**
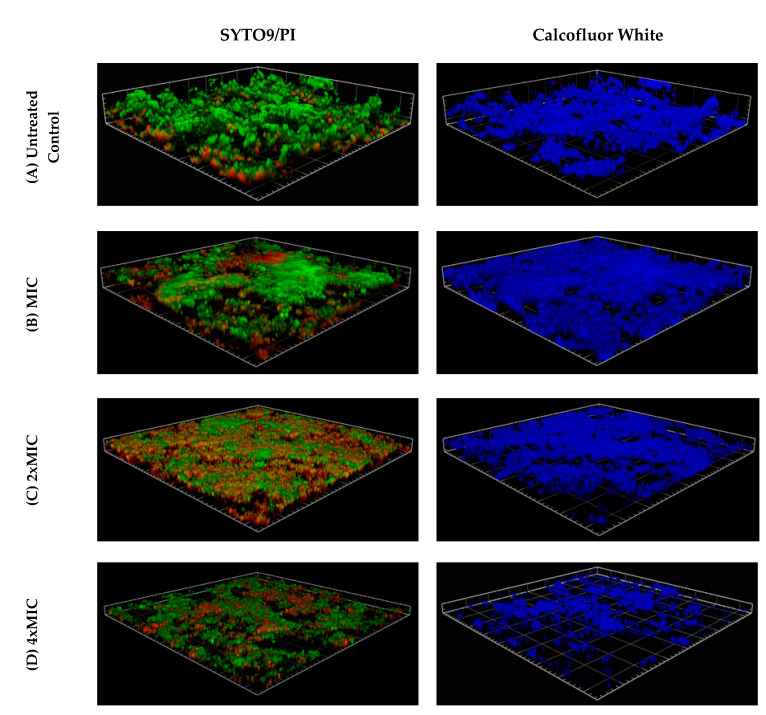
Confocal laser scanning microscopy images of *S. epidermidis* biofilms formed on the surface of aluminum coupons treated with different concentrations of propolis extract. (**A**) The untreated control constituted a 48 h-old biofilm further cultivated for 24 h without the presence of EEPs. (**B**) *S. epidermidis* 48 h-old biofilms were treated with ethanolic extract of propolis (EEPs) at concentrations MIC (128 µg/mL), (**C**) 2x MIC and (**D**) 4x MIC for 24 h. The first column displays bacteria labeled with LIVE/DEAD BacLight SYTO 9 (green) and PI (red) and the second column EPS labeled with CFW (blue). Scale units (grids) are 40 µm in length.

Similarly, confocal microscopy images of the antibacterial assay against *S. epidermidis* biofilms on the surface of nano-smooth aluminum coupons were obtained. On the surface of this material, the *S. epidermidis* ATCC 35984 formed biofilms with complex 3D structure, which is particularly well presented for the untreated control sample (Figure 4A). Effective elimination of bacterial cells required EEP at a concentration of at least 2x MIC (Figure 4B–D). A significant decrease in living cells (green) can be observed for treatments with propolis extracts at a concentration of 2 or 4 x MIC compared to the control (Figure 4A–D). However, the number of cells surviving the treatment in this assay is higher compared to the living cells remaining in the treated biofilm formed on the wells of chambers (Figure 3). On the other hand, a slightly stronger antagonistic effect against the structure of the biofilm was observed on the nano-smooth aluminum surface (Figure 4A–D). Unfortunately, even at the concentration of 4x MIC, biofilms remained partially undisturbed and effectively protected the living cells of *S. epidermidis* ATCC 35984 attached to the coupon’s surface. In this assay, an evident decrease in the number of dead (brown) bacterial cells on the surface of the coupon treated with propolis at a concentration of 4x MIC compared to 2x MIC is also noticeable. It is probably the result of washing away dead cells, which were less closely associated with EPS components with 0.9% NaCl, during the procedure of staining.

## 3. Discussion

According to the U.S. Center for Disease Control (CDC) report, two-thirds of the major bacterial infections reported are due to resistant biofilms, which significantly causes a global burden on human health [21]. It has long been recognized that biofilms increase resistance to antimicrobial action from both external agents, such as antibiotics and internal agents, such as antimicrobial peptides (AMPs), of the innate immune system [22]. As it was mentioned above, staphylococci growing in the form of biofilms exhibit 10 to 1000 times higher antibiotic resistance compared to planktonic cells [10,11]. As biofilm infections significantly contribute to patient morbidity and substantial healthcare costs, novel, effective, and safe strategies to treat these infections are urgently required. The staphylococci are known as etiological factors of two categories of infections: serious, life-threatening systemic infection (e.g., endocarditis) and topical—skin and soft-tissue infections (SSTIs). The SSTIs, however, are not as dangerous as systemic infections and are much more common. In our opinion, the treatment of these infections with alternative (no antibiotic) agents such as plant or bee propolis extracts, essential oils, or honey is promising but is still an underestimated approach. Application of these agents for therapy of systemic infections is problematic because of unknown chemical compositions which may significantly vary between samples. The composition of natural plant products depends on geographical differences, season-to-season variation, weather conditions, or variety of plants used. The outcomes of our previous investigation revealed high efficiency of ethanolic extracts of propolis produced in Polish apiaries in the eradication of *S. aureus* biofilm, with the same values of MIC (Minimum Inhibitory Concentration) and MBEC (Minimum Biofilm Eradication Concentration) parameters—64 or 128 µg/mL for the most active samples (EEP12 and EEP20). Quite similar results were obtained for the sample of propolis used for the current study. The extract produced from this propolis characterized by MIC values of 128 µg/mL against four reference strains of staphylococci and nine clinical isolates of *S. aureus*. The value of the parameter of MBEC_50_ of this extract against biofilm formed by the strain *S. epidermidis* ATCC 35984 was also 128 µg/mL. High, in vitro activity of Polish propolis against *S. epidermidis* biofilm was also observed by Wojtyczka et al. In this study, the biofilms formation ability of *S. epidermidis* ATCC 35983 and 10 strains of staphylococci isolated from blood samples were tested in the presence of EEP. The results revealed significant biofilms formation inhibition at EEP concentrations ranging from 0.39 to 1.56 mg/mL [23]. The anti-biofilm activity of Russian Propolis Ethanol Extracts (RPEE) has been reported by Bryan et al. Confocal microscopy images and results obtained in the MTT (Thiazolyl Blue Tetrazolium Bromide) assay indicated the complete inactivation of *S. aureus* and *E. coli* biofilms after 18 h of treatment. However, relatively high RPEE concentrations of 20% were needed to obtain the effect [24]. In further research of the same group, the viability of biofilms was sufficiently reduced by RPEE at a concentration of 2 μg/mL after 40 h of treatment time. The result indicates that the anti-biofilm activity increases with prolonged treatment duration. The authors also demonstrated that treatment with RPEE resulted in cell lysis and proposed cell membrane disruption as a mechanism for the antibacterial action of RPEE. Another interesting observation made by the authors is that removing heavy metal ions from RPEE drastically reduced toxicity to mammalian cells, which would be beneficial to future medical and biomedical applications [25]. The investigation of Akca et al. revealed quite good activity of Turkish propolis against a broad spectrum of oral bacteria, including staphylococci. MIC and MBC values against planktonic cells of *S. aureus* were 8 and 16 µg/mL, measured by broth microdilution assay. A bit lower activity was observed against biofilms formed by the same strain with MIC and MBC values of 128 and 256 µg/mL, respectively [26]. The most active fractions of ethanolic extracts of Brazilian brown propolis at concentrations of 125 μg/mL killed about 93% of *S. aureus* cells within biofilm [27]. El-Guendouz et al. investigated antimicrobial and antioxidant potential of 24 samples of propolis originating from different locations in Morocco. The authors found that the biofilm-forming ability of diverse MRSA strains were inhibited by propolis of the same origin [28]. Moreover, it was observed that propolis extract at the MIC value of 0.36 mg/mL significantly reduced the virulence potential of *S. aureus* ATCC 6538 and the MRSA strains without leading to the development of resistance after continuous exposure. The authors from Romania found that propolis promoted the prevention of *S. aureus* biofilm formation [29]. de Melo Silva et al. proposed the encapsulation of Brazilian red propolis in polymer nanoparticles. Poly(lactic-co-glycolic acid) nanoparticles (PLGA NPs) containing red propolis hydroethanolic extract (2 mg/mL) were produced by an emulsification solvent–diffusion method. The free extract and the one encapsulated in polymer nanoparticles both presented antimicrobial potential, with a minimum inhibitory concentration from 15.6 to 125 µg/mL and from 100 to 1560 µg/mL inhibiting biofilm formation by the *S. aureus* and *Pseudomonas aeruginosa*, respectively [30]. Propolis extracts also effectively eradicated biofilms formed by other dangerous bacteria, such as *P. aeruginosa* [30,31,32] and pathogenic fungi such as *Candida* spp. [33,34,35]. Our analysis of the literature has shown that another important problem that remains unresolved is the selection of appropriate methods for studying biofilm formation and assessing the effectiveness of antimicrobial agents in biofilm eradication. Different research groups use different methods, which makes it very difficult to compare results. A review of the advantages and disadvantages of the currently proposed methods of biofilm eradication has recently been presented by Jaśkiewicz et al. [36]. The results of our previous study, which was performed with a “classical” in vitro assay in 96-well titration plates, suggested that there is no big difference in EEP activity against both planktonic cells and *S. aureus* biofilm, which would be an important advantage of using propolis over antibiotics. This observation is in line with the outcomes of the current investigation. However, in the case of both studies, we found that increasing EEP’s concentration (up to the concentration of 512 or even 1024 µg/mL) did not reduce the viability of *S. aureus* ATCC 29213 and *S. aureus* ATCC 25923 (previous study) as well as *S. epidermidis* cells ATCC 35984 (current research) by more than about 90%. Herein, we confirmed this observation with fluorescent microscopy. The current studies clearly reveal that even at a concentration four times higher than MIC (or MBEC_50_—determined with “classical”—MTT reduction assay), some living bacteria remained in the structure of EPS. As mentioned above, this residual population is still dangerous to the infected host, potentially contributing to recurrent infections. Another important observation when investigating the antibiofilm potential of propolis is that the structure of EPS is weakly affected by components of propolis EEPs. In fact, the structure of EPS remains mostly undisturbed after treatment, which could potentially inhibit the antibacterial potential of propolis. In our opinion, combined therapy composed of EEPs and antibiofilm agents targeting components of the extracellular matrix should lead to the complete eradication of staphylococcal biofilms without a significant increase of EEPs doses. This approach is the subject of our current research.

## 4. Materials and Methods

### 4.1. Bacterial Strains and Media

The antimicrobial activity of ethanol extract of propolis was tested against four reference strains of bacteria: *Staphylococcus aureus* ATCC 25923, *S. aureus* ATCC 29213, *S. epidermidis* ATCC 12228, and *S. epidermidis* ATCC 35984 (American Type Culture Collection, Manassas, VA, USA). The anti-staphylococcal potential of the propolis sample was also investigated against six MSSA, and three MRSA isolates from patients with different infections (Table 2). *S. epidermidis* ATCC 35984, a strongly adherent, slime-producing strain, was employed as a model for biofilm studies. *S. aureus* and *S. epidermidis* strains were routinely grown on Luria-Bertani Agar (LA, Sigma Aldrich, Schnelldorf, Germany) and Brain Heart Infusion Agar (BHA, Becton Dickinson and Company, Franklin Lakes, NJ, USA). The Minimum Inhibitory Concentration (MIC) was determined using a liquid medium—Mueller–Hinton Broth 2 (MHB2, Sigma Aldrich, Poland). For biofilm formation, TBS liquid medium supplemented with 2.5% of glucose was used (Becton Dickinson and Company, Franklin Lakes, NJ, USA).

### 4.2. Propolis Extract Preparation

A propolis sample was collected from the apiary located in the northern region of Poland (Stanisławowo, Pomeranian Voivodeship Poland) between spring and autumn of 2018. The sample was kept under dry storage at ambient temperature in the dark until processing. Five grams of raw propolis were extracted with 50 mL of 70% ethanol. Extraction was carried out in the dark for 100 h at room temperature with gentle agitation (100 rpm). Afterward, the ethanol extract solutions were centrifuged at 6700× *g* and filtrated through a 0.22 µm pore-size filter. The filtrates were evaporated to dryness at 40 °C using a rotary vacuum evaporator. The obtained resinous substance was weighed, and then the working solution of the extract was prepared at a concentration of 81.92 mg/mL in 70% ethanol.

### 4.3. Total Phenolic Content

The total content of the phenolic compounds was determined using a Folin–Ciocalteu method, as previously described [19]. The Folin–Ciocalteu reagent (Merck, Warsaw, Poland) in the volume of 0.5 mL was mixed with 100 μL of the EEPs (5 mg/mL in 70% ethanol), and, after 5 min, 3 mL of 100 g/L solution of Na_2_CO_3_ (*w/v*) was added. Following shaking, the mixture was made up to a volume of 10 mL with ultrapure water and incubated for 90 min at ambient temperature. The absorbance at 720 nm was measured against a blank (mixture of Folin–Ciocalteu reagent, solution of Na_2_CO_3_ and 70% ethanol in ratios as for samples of EEPs) in a 10 mm quartz cuvette. Total phenol content was calculated and expressed as milligrams of gallic acid equivalent (GAE) per gram, using a calibration curve prepared with a fresh gallic acid (Merck, Warsaw, Poland) standard solution (10–2000 mg/L). All measurements were performed in triplicate.

### 4.4. Minimum Inhibitory Concentration

The minimum inhibitory concentration test was performed by the resazurin-based 96-well plate microdilution method, as described previously [19]. Briefly, two-fold serial dilutions of the propolis extract with a final dilution range from 2048 to 8 mg/L were prepared with Mueller–Hinton II (MHB2) medium (Becton Dickinson and Company, Franklin Lakes, NJ, USA). Then, MHB2 with 1:100 (*v/v*) inoculation from cell adjusted to ~10^8^ CFU/mL was combined 1:1 (*v/v*) with a serially diluted propolis and incubated 24 h under static conditions at 37 °C. After incubation, 30 µL of resazurin (Merck, Warsaw, Poland) solution (0.015% in PBS) was added to each well, and cells were incubated for 2 h at 37 °C in the dark. The lowest concentration with no color change (blue resazurin color remained unchanged) was taken as an MIC value.

### 4.5. Biofilm Formation

#### 4.5.1. Inoculum Preparation

The biofilm-producing strain *S. epidermidis* ATCC 35984 was cultivated overnight in 37 °C on the Brain Heart Infusion (BHI) agar plate (Becton, Dickinson, and Company, Franklin Lakes, NJ, USA). At least ten colonies were picked from the plate, transferred to 5 mL of Tryptic Soy Broth (TSB) medium (Becton Dickinson and Company, Franklin Lakes, NJ, USA), and then incubated at 37 °C for 16–18 h with shaking at 220 rpm. By choosing ten colonies, aberrant results due to clonal differences can be avoided [37]. The bacterial culture was then adjusted to an OD of 0.2 at 600 nm in PBS buffer, diluted 1:100 with TSB medium supplemented with glucose (TSBG; 2.5% *w/v*), and vortexed for 1 min. The biofilms were formed for 48 h at 37 °C under static conditions. Eight-well chamber coverslips and nano-smooth aluminum surfaces were used as in vitro biofilm models.

#### 4.5.2. MBEC Assay

The Minimum Biofilm Eradication Concentration (MBEC) assay was carried out according to the procedure described previously [19]. Bacteria culture was prepared as presented above and dispensed into 96-well microplates. The plates were incubated statically at 37 °C for 24 or 48 h to generate biofilms. After the incubation time, a medium was removed, and the 2-fold serial dilutions of propolis in TSBG medium ranging from 1024 to 32 µg/mL were added to the wells and plates were incubated for an additional 24 h at 37 °C. The MBEC50 values were taken as the lowest concentration of propolis that caused the eradication of at least 50% of living cells in comparison to the cells growing in the untreated control—measured as comparison of the ability of living cells to the biotransformation of MTT (3-(4,5-dimethyl-2-thiazolyl)-2,5-diphenyl-2H-tetrazolium bromide) to insoluble in water violet formazan crystals. After the biofilm formation, the inoculum was removed, and the wells of microplate were washed three times with 100 µL of sterile PBS buffer. Subsequently, 75 µL of PBS and 25 µL of MTT solution (0.3% in PBS) were added to the wells. Following 2 h incubation at 37 °C in the dark, the MTT solution was replaced with 100 µL of DMSO for dissolving formed formazan crystals. Absorbance was measured at 540 nm using a Spark^®^ Multimode Microplate Reader (Tecan, Grödig, Austria).

#### 4.5.3. Biofilm Formation in an 8-Well Chamber Coverslip

In addition, 300 µL of bacterial suspension was immediately dispensed into an 8-well µ-Slide (Ibidi; uncoated: #1.5 polymer coverslip, hydrophobic, sterilized) and incubated at 37 °C for 48 h under static conditions. In order to maintain bacterial viability, the medium was changed after 24 h. To this aim, 80% of the medium was carefully aspirated from the corner of each chamber and replaced with a fresh, pre-warmed medium by slowly dispensing the liquid along walls of chambers. The ethanol propolis extract was added into TSBG at concentrations of MIC, 2x MIC, and 4x MIC (128, 256, 512 µg/mL, respectively). Then, the medium was replaced with the TSBG containing propolis or a fresh medium as an untreated control. The biofilm was further incubated for 24 h. After the desired incubation time, the medium was aspirated from the corners of the chambers, leaving a small amount of liquid in each chamber, and the biofilm was rinsed carefully by slowly dispensing 300 µL of 0.9% NaCl along the walls of the chambers as described in the section above. Immediately after rinsing (to prevent the biofilm from drying out), 200 µL of 0.9% NaCl containing a mixture of fluorescent dyes LIVE/DEAD^®^ BacLight™ (Invitrogen, Warsaw, Poland) SYTO 9 and propidium iodide at the concentration recommended by the manufacturer and Calcofluor White (Biotium, Landing Parkway Fremont, CA, USA) at a concentration of 15 µM/mL) was added and incubated for 20 min at ambient temperature in the dark. The dye mixture was then removed, and the biofilm was carefully washed three times, with 200 µL 0.9% NaCl. Finally, 100 µL of fresh 0.9% NaCl were added. Microscopic observation was carried out immediately after staining.

#### 4.5.4. Biofilm Formation on Aluminum Coupons

The nano-smooth aluminum oxide coupons were constructed using high purity aluminum (99.99%, Alfa Aesar, Ward Hill, MA, USA), as described earlier [38]. The biofilm coupons were ultrasonically cleaned for 30 sec in three cycles, which involved cleaning with chloroform, washing with Milli-Q water, and sterilizing with 96% ethanol. In addition, 2 mL of 1:100 diluted bacteria cultures were transferred into glass tubes and incubated statically for 48 h at 37 °C with vertically placed coupons. After a 24 h incubation period, 80% of the growth medium was removed and replaced with fresh TSBG to maintain bacterial viability. After the incubation time, the biofilm was treated with the propolis at different concentrations (as described above), and the biofilm was challenged for an additional 24 h at 37 °C. An untreated control was incubated with a fresh TSBG medium. The coupons with treated biofilms were removed carefully from tubes and rinsed by submersion in a sterile saline solution (0.9% NaCl) three times. Biofilms were stained with LIVE/DEAD^®^ BacLight™ (Invitrogen, Warsaw, Poland) SYTO 9 (6 µM) and propidium iodide (30 µM), and the EPS was labeled with Calcofluor White (15 µM) (Biotium, Landing Parkway Fremont, CA, USA). The biofilm coupons were placed into 2 mL of 0.9% NaCl containing stain mixture and incubated for 20 min at ambient temperature in the dark. When staining was completed, the coupons were rinsed again in saline solution, gently placed onto a coverslip, and immediately examined via microscopy.

### 4.6. Biofilm Visualization: Confocal Microscopy

Imaging was performed using a Zeiss 710 confocal scanning laser microscope (Zeiss, Oberkochen, Germany) with inverted objectives. Images were taken with a Plan-Apochromat 25 × water objective at a scanning speed of 9 and frame pixels of 512 × 512 (scanning area of 338.4 × 338.4 μm^2^). Bacteria were excited by 488 and 561 nm light and emitted at 491 to 588 nm (green color) for the live cells and 588 to 718 nm (red color) for the dead cells. The excitation wavelength for the EPS was fixed at 405 nm, and the emission wavelengths were set at 410 to 483 nm (blue color). In the “Z-Stack” mode, images were collected every 0.5–1 µm. At least three pictures were acquired in randomly selected places of the biofilm. Three-dimensional images reconstruction of the biofilm matrix was carried out using the Arivis Version4D software (Arivis AG, Munich, Germany).

## 5. Conclusions

The microscopic studies of antibiofilm assays on biofilms formed on the surface of 8-well chamber coverslips and on the surface of nano-smooth aluminum coupons confirmed a promising anti-biofilm potential of EEPs. However, the antibiofilm activity of EEPs is not fully satisfactory in terms of the effectiveness of the elimination of bacterial cells and the disturbing of the EPS structure. In our opinion, a combination of EEPs with agents affecting components of EPS, e.g., proteases, lysines, or enzymes degrading extracellular DNA or PIA, could strengthen the effectiveness of propolis extracts against staphylococcal biofilm. This research is the subject of our current studies.

## Figures and Tables

**Table 1:** Antibacterial activity of ethanol extract of propolis (EEP) tested against reference strains and clinical isolates of *Staphylococcus*.

Bacterial Strains	MIC (µg/mL)
*S. epidermidis* ATCC 35984	128
*S. epidermidis* ATCC 12228	128
*S. aureus* ATCC 29213	128
*S. aureus* ATCC 25923	128
MSSA 4471313	128
MSSA 4475564	128
MSSA 4466686	128
MSSA 4467080	128
MSSA 4467076	128
MSSA 4468505	128
MRSA 45300223	128
MRSA 9944662	128
MRSA 9935169	128

**Table 2:** MSSA and MRSA strains used in this work.

No.	Number	Material	Ward	Antibiogram ^1^
1	4471313	Nasal swab	Intensive care	Pen.–R, Met.–S, Clin.–S, Ery.-S
2	4475564	Nasal swab	Internal	Pen.–R, Met.–S, Clin.–R, Ery.-R
3	4466686	Sputum	Surgical	Pen.–R, Met.–S, Clin.–R, Ery.–R
4	4467080	Nasal swab	Internal	Pen.–R, Met.–S, Clin.–S, Ery.–S
5	4467076	A swab from the ear	Laryngology	Pen.–R, Met.–S, Clin.–S, Ery.–S
6	4468505	Nasal swab	Internal	Pen.–R, Met.–S, Clin.–R, Ery.–R
7	45300223	Blood	Pediatrics	Pen–R, Met–R, Clin–R, Ery–R
8	9935169	Wound	Dispensary	Pen.–R, Met.–R, Clin.–R, Ery.–R
9	9944662	Nasal swab	Dermatology	Pen.–R, Met.–R, Clin.–R, Ery.–R

^1^ –Identification of bacterial isolates and antibiograms were performed by Laboratory of Clinical Microbiology, University Centre for Laboratory Diagnostics, Medical University of Gdańsk Clinical Centre with Vitek2 Biomerieux system; **Pen**.—Penicillin, **Met**—Methicillin, **Clin**—Clindamycin, **Ery**—Erythromycin, **R**—resistance, **S**—sensitive.
